# Genomic advances in the study of the mosquito vector during avian malaria infection

**DOI:** 10.1017/S0031182023000756

**Published:** 2023-12

**Authors:** Irene Hernandez-Caballero, Olof Hellgren, Luz Garcia-Longoria Batanete

**Affiliations:** 1 Department of Anatomy, Cellular Biology and Zoology, University of Extremadura, E-06071 Badajoz, Spain; 2 Molecular Ecology and Evolution Lab, Department of Biology, Lund University, Sölvegatan 37, SE-22362, Sweden

**Keywords:** *Anopheles*, *Culex*, mosquitoes, *Plasmodium*

## Abstract

Invertebrate host–parasite associations are one of the keystones in order to understand vector-borne diseases. The study of these specific interactions provides information not only about how the vector is affected by the parasite at the gene-expression level, but might also reveal mosquito strategies for blocking the transmission of the parasites. A very well-known vector for human malaria is *Anopheles gambiae*. This mosquito species has been the main focus for genomics studies determining essential key genes and pathways over the course of a malaria infection. However, to-date there is an important knowledge gap concerning other non-mammophilic mosquito species, for example some species from the *Culex* genera which may transmit avian malaria but also zoonotic pathogens such as West Nile virus. From an evolutionary perspective, these 2 mosquito genera diverged 170 million years ago, hence allowing studies in both species determining evolutionary conserved genes essential during malaria infections, which in turn might help to find key genes for blocking malaria cycle inside the mosquito. Here, we extensively review the current knowledge on key genes and pathways expressed in *Anopheles* over the course of malaria infections and highlight the importance of conducting genomic investigations for detecting pathways in *Culex* mosquitoes linked to infection of avian malaria. By pooling this information, we underline the need to increase genomic studies in mosquito–parasite associations, such as the one in *Culex*–*Plasmodium,* that can provide a better understanding of the infection dynamics in wildlife and reduce the negative impact on ecosystems.

## Graphical abstract

**Figure:**
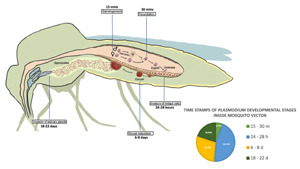

## Introduction

Malaria is an infectious disease caused by a protozoan parasite belonging to the genus *Plasmodium.* These parasites are transmitted through mosquito vectors to a diverse range of vertebrate hosts including mammals like primates, bats and rodents, but also to reptiles and birds (Fricke *et al*., 2010; Schaer *et al*., 2013; Templeton *et al*., 2016). *Plasmodium* species differ in the vector species they are transmitted by, the range of hosts they can infect, their pathogenicity and in their distribution across the world (Levine, 1988; Escalante and Ayala, 1994). In this sense, over 200 morphological species of *Plasmodium* have been formally described based on morphology where 5 of them can infect humans*: Plasmodium falciparum*, *Plasmodium vivax*, *Plasmodium malariae*, *Plasmodium ovale* and *Plasmodium knowlesi* (Sato, 2021). Despite their broad range of infection, some *Plasmodium* species are extremely host specialists such as *P. falciparum*, which infects humans but not African apes that are phylogenetically very close to humans (Liu *et al*., 2010). *P. falciparum* is transmitted by several anopheline species, where *Anopheles gambiae* is one of the most well-known vectors of human malaria (Gouagna *et al*., 2004). This mosquito–parasite association has been widely studied in the last century to bring information to design new strategies to reduce malaria transmission.

Another well-studied group of *Plasmodium* species are those affecting wild birds, i.e. avian malaria parasites (LaPointe *et al*., 2012). Avian malaria encompasses more than 40 morphologically described *Plasmodium* species (Atkinson, 1991) but over 500 different lineages have been described using sequence divergence in the mitochondrial cytochrome b gene (Bensch *et al*., 2004, 2009). These parasites are mainly transmitted by *Culex* mosquitoes (Fonseca *et al*., 2004). Within this mosquito genera, *Culex pipiens* species complex may act as vector for *Plasmodium* species such as *Plasmodium relictum* (Lapointe *et al*., 2010) and *Plasmodium gallinaceum* (Pruck-Ngern *et al*., 2015). The importance of studying *P. relictum* and its association with both its vertebrate and invertebrate hosts relies on the fact that it is one of the most widespread avian malaria parasites in the world (Kazlauskienė *et al*., 2013; Valkiūnas *et al*., 2018). Moreover, this malaria species is responsible for several bird species extinctions (Atkinson and Samuel, 2010) and is currently listed as one of the 100 most dangerous invasive species in the world (Boudjelas *et al*., 2020).

**Figure 1.**
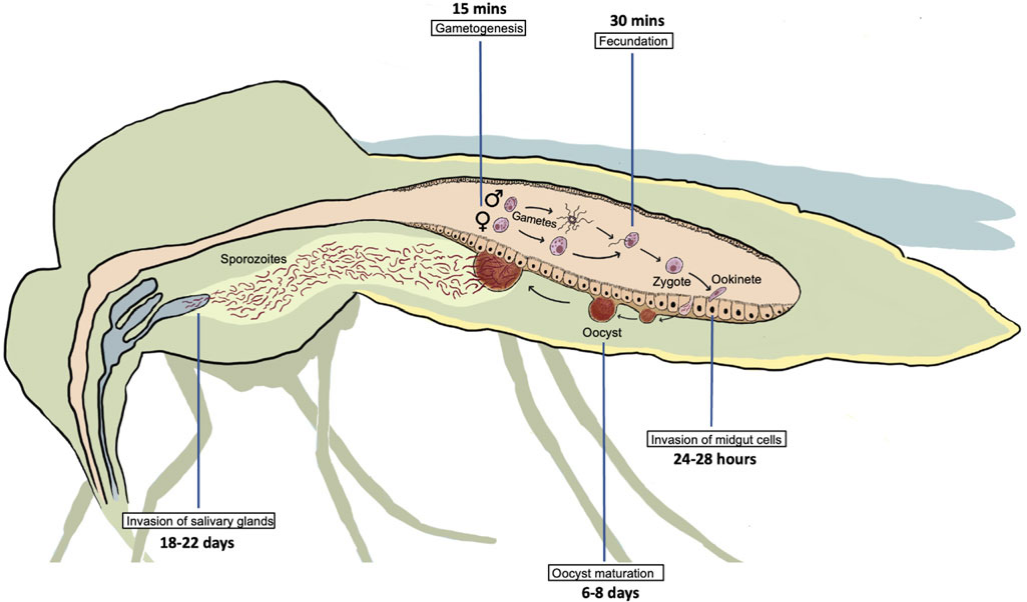
Developmental stages of *Plasmodium* during sexual reproduction inside its invertebrate host. Time points in the figure correspond to time post blood feeding (PBF). Gametogenesis occurs 15 min PBF when ingested gametocytes of *Plasmodium* develop into male and female gametes, followed by fecundation 30 min PBF leading to the production of zygotes, that develop into motile ookinetes. Invasion of the midgut cells by ookinetes takes place between 24 and 28 h PBF. Oocyst maturation takes place between 6 and 8 days PBF followed by release of sporozoites and migration to salivary glands that conclude with their ejection along the saliva into a new vertebrate 18–22 days PBF.

All *Plasmodium* species share a similar but complex life cycle (Votýpka *et al*., 2016) that involves 2 separate hosts: a vertebrate host and a mosquito vector (invertebrate host). Inside each host, the parasite undergoes multiple developmental stages. The life cycle of *Plasmodium* begins in the vertebrate host, when the sporozoites are expelled with the saliva of the female mosquito while is taking a blood meal. Inside the vertebrate host, the parasite undergoes different developmental stages that conclude with the production of gametocytes. The next step is the transmission of gametocytes to an invertebrate host (Fig. 1), which is achieved when a female mosquito feeds on infected blood. The ingested gametocytes of *Plasmodium* develop into male and female gametes in the midgut lumen. Inside the mosquito vector, the parasite reproduces sexually (Bennink *et al*., 2016), and the fertilized gametes produce zygotes, the only diploid stage of the parasite, which develop into motile ookinetes that invade the epithelium of the midgut cells in the mosquito to reach its basal side. Ookinetes then develop into oocysts that produce several sporozoites, which mature in a period that varies depending on the *Plasmodium* species. Once the maturation period is over, the midgut sporozoites are released into the haemolymph and migrate to the salivary glands where they are ejected along the saliva into a new vertebrate to start a new life cycle (Vaughan, 2007).

The family Culicidae comprises several genera, including *Culex* mosquitoes that diverged from *Anopheles* during the early Jurassic period (~160–200 million years ago, da Silva *et al*., 2020; Lorenz *et al*., 2021). They are species of medical and veterinary importance that act as vectors for shared pathogen groups, such as *Plasmodium* spp. Most genomic studies are currently focused on *Anopheles* species since *An. gambiae* genome was completely sequenced more than 20 years ago (Holt, *et al*., 2002), and since then it has been widely used to investigate mosquito DNA expression patterns to *Plasmodium* infection. The genome of *Culex quinquefasciatus* was reported more recently (Arensburger *et al*., 2010), showing great differences in genome size and in the total number of genes between the 2 mosquito species. In this sense, the genome of *An. gambiae* is smaller (278 Mb) than *Cx. quinquefasciatus* genome (579 Mb) and, therefore, the number of annotated genes is slightly bigger in *Cx. quinquefasciatus* (Severson and Behura, 2012). However, although the information of the genomes of both mosquito species is available since long time ago, there is an important knowledge gap concerning gene expression in response to *Plasmodium* infection in the *C. pipiens* complex.

Here, we extensively review the current knowledge on the regulation of key genes of the avian mosquito vector *Cx. quinquefasciatus*, relevant during *P. relictum* infection. We also aim to compare the activation of genes expressing important immune and metabolic pathways during *Plasmodium* infections between the human and avian malaria mosquito vectors during *Plasmodium* infection, *Anopheles* and *Culex*, respectively, to highlight the limited number of genomic studies focusing on *Culex*. We further describe problems that may limit genomic research in *Plasmodium*-infected vectors, such as the time elapsing since the mosquito takes a blood meal to sampling point, the proportion of malaria-infected mosquito cells (parasitaemia), the variability of vector gene expression among collected tissues and specific parasite–vector associations.

## Materials and methods

**Figure 2.**
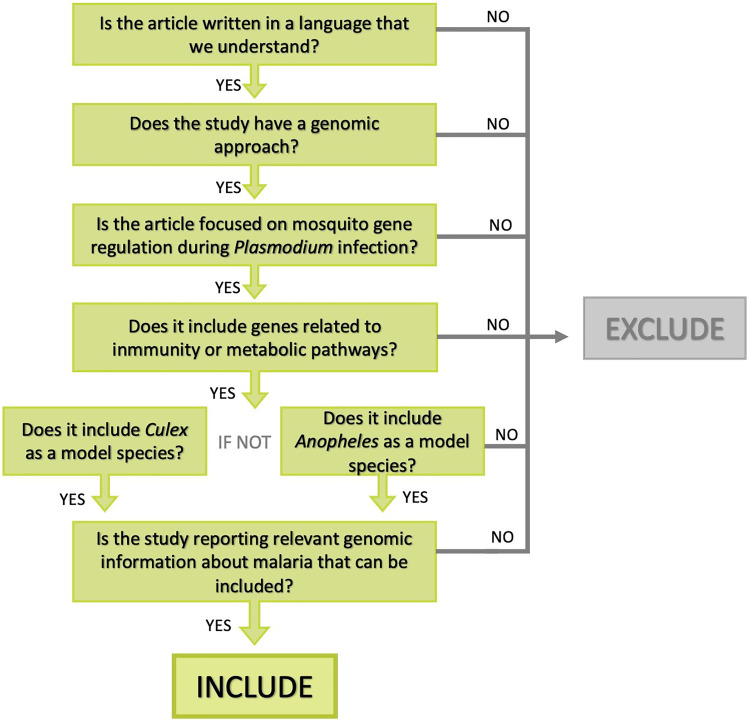
Decision tree based on PRISMA framework. Inclusion/exclusion criteria were used to filter studies focused on genes involved in important immune and metabolic pathways in *Anopheles* and *Culex* during *Plasmodium* infection.

Our literature search was conducted in September 2022. Initial title, keyword, and abstract screening was performed using the items for systematic review and meta-analysis established in PRISMA (Moher *et al*., 2009) modified for Ecology and Evolution, PRISMA-Eco Evo (O'Dea *et al*., 2021). A systematic search on the available literature on genomic analysis of *Anopheles* and *Culex* mosquitoes infected with *Plasmodium* was performed on the *Web of Science* (WoS) database. The search was conducted in English. We included articles published between 2002 and 2023 years (see Supplementary Table 1) searching with specific Booleans (see below). The search string comprised 2 substrings. The first substring targeted mosquito genomic changes during infection using the following Boolean search keywords [Genom* AND transcriptom* Vector AND mosquito AND Anopheles OR Culex AND malaria AND association AND infection]. We retrieved 358 articles on WoS. The second substring aimed at the evolution of *Anopheles* and *Culex* linked to genomic and transcriptomic analysis during infection using the following Boolean search keywords [Genom* AND transcriptom* Vector AND mosquito AND Anopheles OR Culex AND malaria AND evolut*]. We retrieved 531 articles on WoS. After the removal of duplicates between the 2 substrings, we obtain a subtotal of 635 articles that were screened at title, keyword and abstract stage. Five hundred and sixty-eight articles were excluded for further analyses because they were not related to our aim of study. In consequence, full-text of the remaining 67 articles showing genomic and transcriptomic analysis of mosquitoes infected with *Plasmodium* were screened in a decision tree containing our inclusion/exclusion criteria, following the guidelines for systematic search and study screening for literature reviews in Ecology and Evolution proposed by Foo *et al*. (2021) (Fig. 2). Twenty-three studies met all our inclusion criteria. All these studies examined genes involved in important immune and metabolic pathways in human or avian malaria mosquito vectors *Anopheles* and *Culex* during natural and non-natural *Plasmodium* infection.

## Current knowledge on the genomics of mosquito during malaria infections

*An. gambiae* has been used in many research studies to gain genetic insights that might help prevent and eradicate malaria. In this sense, transcriptomic analyses are a useful tool to understand the role of the mosquito in *Plasmodium* transmission, as they bring information about the regulation of RNA expression during an exact moment of a specific event during the parasite infection (Domingos *et al*., 2017). There is a huge number of studies providing information about fundamental aspects of *An. gambiae* gene expression during both non-natural (i.e. when infection experiments uses a combination of parasite and vector species that have not been observed in the wild) and natural malaria infection (Dong *et al*., 2006; Baton *et al*., 2009; Mead *et al*., 2012; Biryukova *et al*., 2014; Ruiz *et al*., 2019). Thanks to these studies the information regarding immune and physiological response linked to various parasite developmental stage is quite broad. However, transcriptomic studies focused on the *Culex* complex are still scarce, even during *P. relictum* infection.

### Genes involved in immune responses

When a mosquito bites a non-infected vertebrate host, the expression of genes related with several biological processes important for reproduction and survival such as egg production or cell homoeostasis is affected (Bryant *et al*., 2010). Infected blood that contains malaria sporozoites activates different mechanisms linked to immune response (Luckhart *et al*., 1998) or cell apoptosis (Ahmed and Hurd, 2006). In dipterans, there are 3 genes' categories involved in the innate immune response against *Plasmodium* which regulation is well described in *An. gambiae*: (i) recognition proteins of pathogen's components (Dong *et al*., 2006; Gendrin *et al*., 2016), (ii) components of signalling pathways related to the modulation, amplification, and transduction of cell signals (Chen *et al*., 2012) and (iii) antimicrobial peptides (AMPs), complement factors and enzymes (Dixit *et al*., 2008; Clayton *et al*., 2014).

Mosquitoes, like the rest of invertebrates, rely on innate immunity as their only defence system (Christophides *et al*., 2004). Immune response occurs in several tissues of the mosquito: the midgut epithelium, lumen, haemolymph and within the salivary glands (Osta *et al*., 2004). When *Plasmodium* (or other infectious microorganisms) infect the mosquito, 2 main responses might be activated against the parasites: the humoral and cellular responses. Humoral response is formed by 3 main immune pathways: Toll, Imd and JAK/STAT (Dimopoulos *et al*., 1997; Tikhe and Dimopoulos, 2021). These immune pathways include different immune cascades that conclude with the transcriptional regulation of mechanisms that aims to clear the parasite from the vector (Dong *et al*., 2020). In cellular response, different immune components like enzymes (Dong *et al*., 2006) or specific cells like haemocytes physically isolate and destroy the parasite (Clayton *et al*., 2014). Interestingly, these immune responses reducing *Plasmodium* parasitaemia take place in 3 events of *Plamodium* life-cycle inside the mosquito vector: (i) the ookinete maturation, also limited by molecules from vertebrate host and digestive molecules from mosquito secreted into the bloodmeal (Sinden *et al*., 2004), (ii) the invasion of the midgut by ookinetes and (iii) the sporozoite migration through the haemocoel to the salivary glands (Ghosh *et al*., 2001; Shahabuddin and Costero, 2001; Sinden *et al*., 2004). These mechanisms, are attributed to haemocyte-mediated immune responses (Frolet *et al*., 2006) that activate genes in the midgut of the mosquito (Dong *et al*., 2006).

Genomic studies in *An. gambiae* show a variety of results depending on the vector–parasite association. Most genomic studies of *Anopheles* mosquitoes have explored non-natural parasite–vector associations, such as, *An. gambiae* infected with a rodent malaria parasite (*Plasmodium berghei*). In genomic studies of immune response, rodent malaria parasite *P. berghei* is commonly used to experimentally infect *An. gambiae* for the identification of key genes for the innate immune system (Baton *et al*., 2009; Raddi *et al*., 2020). Nonetheless, to get a more realistic figure of how *Plasmodium* parasites trigger immune responses in the mosquito, such associations should be better assessed in natural parasite–vector associations, as many responses both in the vector and in the parasite might have co-evolved over long periods of time. For example, the infection of *An. gambiae* with the non-natural parasite *P. berghei* and *An. gambiae* led to a greater activation of the Toll pathway during the mosquito immune response (Clayton *et al*., 2014). On the contrary, the infection of *An. gambiae* with its natural parasite *P. falciparum* induced a greater activation of genes involved in the Imd pathway (Garver *et al*., 2009; Dong *et al*., 2011, 2006). Thus, studies focused on natural parasite-mosquito associations are essential for a better understanding of how malaria impacts on their vector gene expression. Up to date, transcriptomic studies analysing *Cx. quinquefasciatus* gene expression using natural parasite association provide valuable information about immune response in other parasite–vector associations apart from *Anopheles*. For example, Garcia-Longoria *et al*. (2022) have recently presented the first transcriptomic study analysing the effect of *P. relictum* infection on *Cx. quinquefasciatus* immune response. In this natural parasite–vector association, infected mosquitoes showed a greater activation in the Toll pathway, followed by Imd pathway, through the up regulation of several receptors, translation factors and effectors. They also found up regulation of genes related to cellular response in melanization cascade elements, CLIP-domain serine proteases and serpins genes, indicating that these processes may play an important role in the defence against *P. relictum*. However, other natural avian malaria parasite–vector associations, such as the one between *Culex* species and *P. relictum* lineages, have received less attention, and further studies are required to need to verify the up or down regulation of immune pathways and cascades over the avian malaria infection.

### Genes involved in metabolic pathways

During malaria infection, mosquitoes exhibit several changes in the expression of its genes involved in metabolism that are key to *Plasmodium* development (Vlachou *et al*., 2005). For instance, malaria parasite gametogenesis is triggered by a molecule called xanthurenic acid (XA) (Garcia *et al*., 1998; McRobert *et al*., 2008) which is an intermediate metabolite of tryptophan in the mosquito (Billker *et al*., 1998). This molecule induces intracellular rise in Ca^2+^ concentration to activate a protein kinase within the parasite, that regulate gametogenesis (gametocyte differentiation into male and female gametes) and *Plasmodium* transmission (Billker *et al*., 2004; Brochet and Billker, 2016). Specifically, in *P. berghei* (Billker *et al*., 2004) and in *P. falciparum* (McRobert *et al*., 2008) this intracellular Ca^2+^ is essential for the exflagellation process.

Likewise, Guttery *et al*. (2013) demonstrated in laboratory conditions that environmental Ca^2+^ has an impact on *P. berghei* sexual development. They genetically modified PbCAX gene, a *P. berghei* Ca^2+^/*H^+^* exchanger, which is important to maintain Ca^2+^ homoeostasis. As a result, parasites with experimentally disrupted genes failed to produce zygotes. Moreover, this process could be reversed *in vitro* by removing environmental Ca^2+^. They concluded that PbCAX is essential to tolerate Ca^2+^ within the ionic environment of the mosquito midgut, and ultimately, for ookinete development and differentiation within the mosquito. Interestingly, Ferreira *et al*. (2022) experimentally infected wild-caught mosquitoes from the Hawaiian Islands and simulated natural conditions to reflect more reliable effects of the *P. relictum–Cx. quinquefasciatus* association. They showed important differences between infected and uninfected mosquitoes in the expression of genes related to calcium transportation 24 h and 5 days PFB. More specifically, they found that infected mosquitoes had higher expression levels of genes involved in calcium transportation or binding at 24 h PBF. Also, biological process related with endoplasmic reticulum calcium ion homoeostasis was significantly higher at 5 days PFB in infected mosquitoes (Ferreira *et al*., 2022).

Glucose is the main source of energy not only for the mosquito but also for the malaria parasite (Liu *et al*., 2013). Blood stages of malaria parasites are dependent on glucose catabolism components such as adenosine triphosphate to obtain their main source of energy (Kirk *et al*., 1996). Meireles *et al*. (2017) demonstrated the role of glucose levels in the successful hepatic infection of *P. berghei,* where glucose levels below a standard medium concentration led to failed infection. In addition, they showed that there is an increase in glucose uptake *via* the GLUT1 transporter (class I facilitative glucose transporter expressed in liver cells) in *P. berghei*-infected hepatic cells.

Following this idea, Wang and Wang (2020) examined the function of the glucose transporter Asterglut1 in the non-natural association *Anopheles stephensi–P. berghei.* They found that the knockdown of the glucose transporter genes significantly increased the glucose level in the midgut of the mosquito prior to blood feeding and increased *P. berghei* infection, hence suggesting that Asteglut 1 participate in the defence against malaria infection. Ferreira *et al*. (2022) evaluated the gene-expression response of *Culex* mosquitoes exposed to *Plasmodium* infection in a natural parasite–vector association, reporting a lower expression level in infected mosquitoes compared to control in a gene involved in glucose metabolism (6-phosphogluconate dehydrogenase) during ookinete invasion (24 h post feeding), and in an unknown sugar transporter gene (CPIJ008946) 10 days post feeding.

The same level of metabolic importance is attributed to solute carriers transporting ions helping to maintain ionic homoeostasis (Hirata *et al*., 2012). Recent studies have shown the importance of these solute carriers in both natural and non-natural parasite- vector associations. In *An. gambiae* experimentally infected with rodent malaria (*P. berghei*), infected mosquitoes showed an upregulation of solute carrier genes involved in cell homoeostasis in the salivary glands 18 days post blood feeding (PBF) (Couto *et al*., 2017). Moreover, the infection of *An. gambiae* mosquitoes with *P. berghei* with knocked down solute carrier genes induced a reduction of the number of sporozoites in the salivary glands and increased mosquito death rate (Couto *et al*., 2017). Accordingly, in a natural parasite–vector association, *Cx. quinquefasciatus* infected by *P. relictum* showed significantly higher expression of several anion and ion transporter genes 5 days PBF (Ferreira *et al*., 2022). These results might indicate that *Plasmodium* may exploit *An. gambiae* (Couto *et al*., 2020) and *Cx. quinquefasciatus* cellular mechanisms to obtain resources to maximize its reproduction and transmission.

## Problems related to work with mosquito–parasite association through transcriptomics

Parasitaemia levels (the proportion of malaria infected cells inside an organism) might affect host transcriptome response (Videvall *et al*., 2020) as differences in the parasite load may harm and affect mosquitoes at a different scale. Accordingly, organisms with higher parasitaemia usually show higher amounts of differentially expressed genes compared to those with lower parasitaemia. In this sense, a previous study has suggested that a strong response to infection is accompanied by high parasitaemia rates (Videvall *et al*., 2020). For example, in the case of *Culex* mosquitoes, 2 recent studies have shown different transcriptomic responses probably due to mosquitoes harbouring different levels of parasitaemia caused by 2 different avian malaria strains (*P. relictum* pGRW04 and pSGS1) (Ferreira *et al*., 2022; Garcia-Longoria *et al*., 2022). Garcia-Longoria *et al*. (2022) experimentally infected *Culex* mosquitoes with *P. relictum* pSGS1, achieving fairly high levels of parasitaemia and, a significant amount of up-regulated immune genes. However, Ferreira *et al*. (2022), analysed the gene expression of *Culex* mosquitoes naturally infected with *P. relictum* pGRW04, showing a low parasitaemia in these mosquitoes and a low number of significant genes responding to the infection.

Another weakness when analysing transcriptomic in insects during mosquito–parasite associations is limitation of tissue sampling, which can lead to tissue-biased expression (Baker *et al*., 2011). *Plasmodium* infection in mosquitoes is quite restricted to specific tissues where sporozoites are mainly detected in salivary glands, ookinetes in the gut walls and gametes inside the gut (Valkiūnas, 2005). This differentiation potentially might complicate the detection of mosquito readings since the detection of transcriptomic signals can be masked by mosquito tissues that are more abundant in the sample. This is an important issue to deal with because it can lead to false negatives and, therefore, to lose information about differentially gene expression.

An important caveat in the study of mosquito–parasite association is the lack of a well assembled and annotated genome. In the case of *Culex* mosquitoes, previous studies have detected around 20% of uncharacterized genes in their analyses (see Ferreira *et al*., 2022, but also see Garcia-Longoria *et al*., 2022), thus highlighting the need to improve the annotation and gene prediction of the *Culex* assembled genomes. Nevertheless, this is not the case for genomes related to *Anopheles* family. Since the publication of *An. gambiae* genome by Holt *et al*. (2002) several updates from this genome have been made resulting in high-quality reference genome where a high number of annotated genes can be detected when analysing transcriptome response (Sharakhova *et al*., 2007; George *et al*., 2010; Kingan *et al*., 2019).

**Figure 3.**
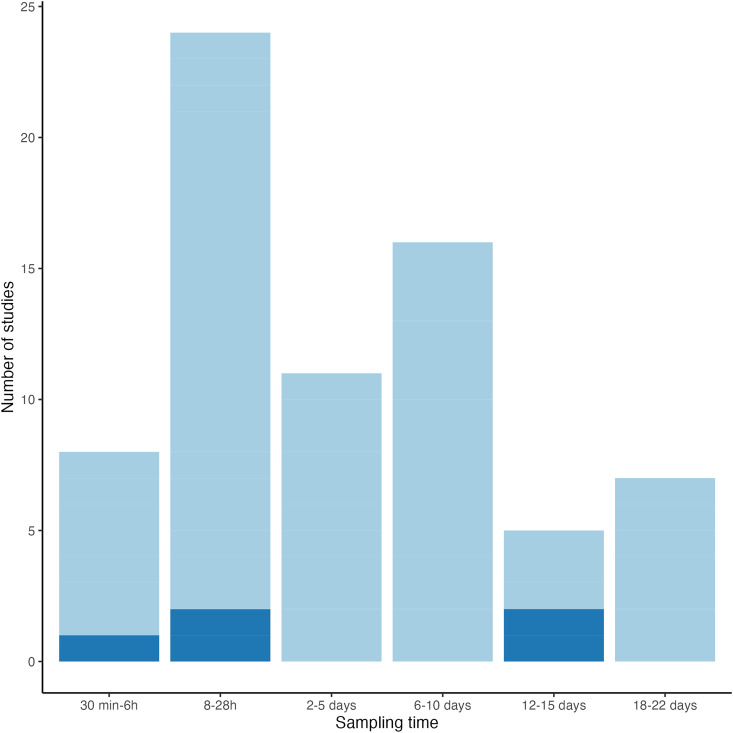
Number of studies analysing gene expression at different times post blood feeding (PBF). *X* axis shows the number of articles focusing a specific sampling time interval and *Y* axis shows the different times of sampling. Single time points are shown in dark blue and arrangement of time points are shown in light blue.

Time points of sampling PBF is another crucial aspect in transcriptomic research, and it is directly related to malaria parasite development inside the mosquito (Fig. 3). The duration and timing of the different stages of the malaria life cycle differ between *Plasmodium* species, and it is determined by factors such as internal mosquito temperature and pH (Beier, 1998). The arrangement of time points in these genomic analyses include a few minutes and hours after the blood meal was taken to several days after blood meal ingestion. There is a reduced number of studies analysing vector gene expression on a single time point in comparison to those focused on a range of different time points. In this sense, only 5 studies analysed the gene expression on a single time point PBF, whereas 18 studies were done using and an arrangement of different time points PBF (Fig. 3; See Supplementary Table 1).

Early time points PBF (from 30 min PFB to 12 h PFB) are used for detecting the initial effect of *Plasmodium.* For example, in *Cx. quinquefasciatus* it has been shown that only 2 receptors of Toll pathway (CPIJ019764, CPIJ018343) were significantly up-regulated at 30 min PBF, but there were not Toll transcription factors expressed at this time point (Garcia-Longoria *et al*., 2022). Most studies use a range from 18 to 28 h PBF (Dimopoulos *et al*., 2002; Vlachou *et al*., 2005; Xu *et al*., 2005; Baton *et al*., 2009; Mead *et al*., 2012; Alout *et al*., 2013; Nsango *et al*., 2013). Specially, 24 h PBF is a fairly used time point in genomic studies, because it is the time where *Plasmodium* ookinetes invade the epithelium of midgut cells and reach its basal side (Osta *et al*., 2004). Accordingly, Garrigós *et al*. (2023) found that at 24 h PBF, *P. relictum* induced the expression of spätzle gene (CPIJ006792), a ligand of Toll receptors. This initial stage is then followed by the development of oocyst into sporozoites for 6–10. A large amount of transcription factors and its inhibitors are expected to be regulated during this period. In line with this idea, a striking up regulation of Toll receptors like Dorsal transcription factor within the Toll pathway (CPIJ002469) has been shown at 8 days PBF in *Cx. quinquefasciatus*, whereas its inhibitor the cactus protein CPIJ004774), is down regulated at this stage (Garcia-Longoria *et al*., 2022). Finally, a reduced number of studies use the range between 18–22 days (Xu *et al*., 2005; Couto *et al*., 2017; Zhang *et al*., 2017; Carr *et al*., 2021; Garrigós *et al*., 2023), which is the stage related to sporozoite migration to the salivary glands (Amino *et al*., 2008). A smaller number of transcription factors and receptors are expected to be expressed 22 days PBF. According to this, Garcia-Longoria *et al*. (2022) reported no differences in the expression of both the toll transcription factors and its inhibitor proteins between *Plasmodium*-infected and uninfected *Culex* mosquitoes at 22 days PBF.

## Discussion

Metabolic and immune response of mosquitoes during *Plasmodium* infection affects parasite fitness, by limiting its capacity to survive within the host, to reproduce and to be transmitted into new hosts. Since *Plasmodium* reproduction and transmission is linked to mosquito derived molecules such as XA, the expression of genes related to tryptophan metabolism could be a targeted by the parasite to increase its fitness. Following this idea, Ferreira *et al*. (2022) suggested that since calcium is essential for *Plasmodium* ookinete motility and gametogenesis (Luckhart *et al*., 1998), an enhanced expression of mosquito calcium transporters could supply malaria parasites with Ca^2+^ to facilitate midgut invasion. Alternatively, an enhanced expression of calcium transporters (as shown in Ferreira *et al*., 2022) could be a mosquito response to malaria infection, because high levels of environmental Ca^2+^ can be a threat to parasite homoeostasis and limit *Plasmodium* development (Guttery *et al*., 2013). Nevertheless, there is not an agreement about the real effect of mosquito-derived calcium and the implication of mosquito transporters during *Plasmodium* development within the mosquito.

Also, while most of the studies focused on metabolic components relevant in malaria transmission are primarily focused on *Anopheles* (Adedeji *et al*., 2020), there is little information about the role of metabolic gene regulation during avian malaria infection in *Culex*. Whether these components of metabolism might play a role in the success of avian malaria infection in invertebrate vector requires further investigation.

Natural and non-natural *Plasmodium*-vector associations have significantly different profiles for immune activation (Sreenivasamurthy *et al*., 2013). As showed in previous sections, non-natural associations in *Anopheles*–*Plasmodium* activate the Toll pathway while in natural associations in *Anopheles*–*Plasmodium* takes place to the Imd pathway activation (Garver *et al*., 2009; Dong *et al*., 2011; 2006). Additionally, natural *Culex*–*Plasmodium* associations take place to the activation of both immune pathways (Garcia-Longoria *et al*., 2022). Interestingly, both immune pathways are supposed to be established early in the evolution of metazoa (Hoffmann *et al*., 1999); Imd pathway is supposed to be more effective towards *Plasmodium* (Meister *et al*., 2005), whereas Toll pathway is more specific towards bacteria and fungi in mosquitoes (Tikhe and Dimopoulos, 2021). However, differences in immune gene expression depending on the mosquito–parasite association are largely unknown among vector species. It could be hypothesized that these differences between pathway activation in natural and non-natural associations between *Anopheles* and *Culex* may be linked to differences in parasitaemia, or disparities in co-evolutionary history between hosts and parasites, among others. Nonetheless, more studies comparing side-by-side natural and non-natural mosquito vector–malaria parasite associations need to be explored.

Regarding the problem of parasitaemia and transcriptome response, the different outcomes in gene expression showed by Garcia-Longoria *et al*. (2022) and Ferreira *et al*. (2022) highlight that parasitaemia is an important limitation when analysing transcriptomic response during a host–parasite association. The different levels of parasitaemia achieved during the infection of the mosquito *Cx. quinquefasciatus* by 2 closely related *P. relictum* parasites (pSGS1 and pGRW04) might explain differences in transcriptomic responses between these 2 studies. Future researchers should take these restrictions into account during the experimental design and try to go deeper in the effects that parasitaemia caused on transcriptomic results depending on the avian malaria strain.

The limitation imposed by the potential bias in gene expression determined by the origin of the collected tissue should be addressed in further studies. In this sense, it becomes essential to differentiate the RNA expression analyses depending on the tissue through a prior dissection of the mosquitoes and sequencing specific tissues as a whole. However, including a dissection step into the analysis might also affect the observed gene regulation due to the possibility that the RNA will degenerate during the process. In avian malaria–vector associations, tissue-specific research is still a pending task, but it has been explored in several studies in human malaria (Dixit *et al*., 2009; Sreenivasamurthy *et al*., 2017). Previous studies in *Anopheles* mosquitoes focused on specific tissues have been able to detect not only parasite strategies for avoiding mosquito immune system (Xu *et al*., 2005) but also to differentiate gene expression of individual sporozoites through single-cell RNA sequencing (Ruberto *et al*., 2021).

Perhaps, the key stone to understand mosquito gene regulation during malaria infection would be to increase the number of annotated genes. Future improvements in *Culex* genomes would help to further understand how these mosquito families respond to malaria infection and the degree to which they have evolved immunity along different or similar evolutionary routes.

Finally, sampling time PBF should be considered a crucial issue in genomic studies, since different time points PBF are linked to different outcomes in expression patterns. Studies using a wide arrangement of time points PBF are useful to understand the changes of mosquito gene expression along the parasite development, and could be important for designing new tools for malaria control. Future studies should analyse vector gene expression over a wider range of time points PBF to extend the knowledge about malaria in early and long-term effects.

Although early time points are the less studied, a recent study has reported that a great number of genes are differentially expressed in *Culex* at 24 h PBF (Garrigós *et al*., 2023). After this time point, there is a substantial reduction in the number of genes expressed by the mosquito over time. Studies focusing on initial stages of infection could bring important information about gene expression during these early steps of infection, and even clues to examine potential adaptive parasite manipulations on the invertebrate host.

## Concluding remarks

Genomic studies are an essential tool to understand the dynamic of vector borne diseases like malaria. It is crucial to reveal the regulatory genomic changes in vectors during parasite development, as it might lead to key information that can be used to prevent the spreading of the disease. Many genomic studies have been centred on natural and to a large degree non-natural associations between *Plasmodium* species and the mosquito vector *An. gambiae*. Regarding the human system, more studies based on natural parasite–vector associations *P. falciparum* and *An. gambiae* are needed to clearly identify the coevolution between these 2 organisms. The information focusing on the natural association between *Plasmodium* and other genera of mosquito vectors, such as *Culex*, is even scarcer. A very representative example is the *C. pipiens* complex transmitting avian malaria parasites, where only 3 natural *Culex–Plasmodium* associations have been explored. Specifically, between 1 mosquito species, *Cx. quinquefasciatus* and 2 *P. relictum* lineages (pGRW04, pSGS1) (Ferreira *et al*., 2022; Garcia-Longoria *et al*., 2022) and 1 *P. cathemerium* lineage (PADOM02) (Garrigós *et al*., 2023), which have shown differences in transcriptomic responses. Future research should use up-to-date RNA-sequencing techniques and optimized sampling protocols to efficiently explore the effects of natural associations in *Culex* gene expression during different stages of parasite development, and compare their results with those obtained from natural and non-natural vector–parasite associations in human malaria. By doing this, genomic differences and similarities between *Anopheles* and *Culex* mosquitoes infected with *Plasmodium* parasites would help for a better understanding on how these 2 distantly related vector species respond over the infection, and also on how the parasite might manipulate vector gene expression for its own benefit. This understanding would be useful for the development of new molecular techniques for malaria control.

## Supporting information

Hernandez-Caballero et al. supplementary material Hernandez-Caballero et al. supplementary material

## Supplementary material

The supplementary material for this article can be found at https://doi.org/10.1017/S0031182023000756
